# A Narrative Review on the Impact of ENDS Use on Clinical, Inflammatory and Microbiological Periodontal-Related Parameters

**DOI:** 10.3390/dj14070452

**Published:** 2026-07-19

**Authors:** Sun Ha Park, Jun Yaung, Sheila Yaghmai, Beatriz Bezerra

**Affiliations:** 1 School of Dentistry, University of California, Los Angeles, CA 90095, USA; sunhapark19@g.ucla.edu (S.H.P.); jun.yaung@g.ucla.edu (J.Y.); 2 Section of Interdisciplinary Dentistry, School of Dentistry, University of California, Los Angeles, CA 90095, USA; syaghmai@dentistry.ucla.edu; 3 Section of Periodontics, School of Dentistry, University of California, Los Angeles, CA 90095, USA

**Keywords:** electronic nicotine delivery systems, periodontal disease, oral microbiome, inflammatory markers, vaping, e-cigarettes

## Abstract

**Background/Objectives:** The increasing prevalence of electronic nicotine delivery systems (ENDS) has prompted questions regarding their effects on periodontal health. While the detrimental impacts of cigarette smoking are well-established, the periodontal implications of ENDS use remain poorly understood. This narrative review aimed to evaluate current evidence comparing periodontal parameters, inflammatory markers, and oral microbiome composition among ENDS users, cigarette smokers, and non-smokers. **Methods:** A structured literature search was conducted in PubMed (January 2009–May 2026) following a PICO question: “What is the impact of ENDS, compared to cigarette smoking and non-smoking, on periodontal health?” Clinical studies reporting on periodontal health parameters (plaque index, bleeding on probing, probing depth, and clinical attachment loss), inflammatory markers (e.g., IL-1β, IL-6, TNF-α), and microbial composition were included. Due to methodological heterogeneity across studies, meta-analysis was not feasible, necessitating narrative synthesis. **Results:** Across studies, clinical periodontal findings in ENDS users were heterogeneous and did not consistently resemble those of either cigarette smokers or non-smokers. In contrast, ENDS use was consistently associated with alterations in inflammatory profiles and oral microbiome composition, characterized by selective changes in specific inflammatory cytokines rather than uniform inflammatory upregulation, as well as shifts towards microbial communities linked to periodontal inflammation. Notably, these biological alterations were not uniformly reflected in traditional clinical periodontal indices. **Conclusions:** ENDS use is associated with heterogeneous clinical findings and inflammatory and microbial alterations that suggest ENDS is a distinct exposure rather than a predictable intermediate between smoking and non-use. This review, to our knowledge, is the first to integrate clinical, inflammatory, and microbiome outcomes and highlights the limitations of relying solely on clinical parameters to assess the impact of ENDS on periodontal health. It also underscores the need for well-designed longitudinal studies to clarify the periodontal implications of ENDS use.

## 1. Introduction

Electronic nicotine delivery systems (ENDS), commonly known as e-cigarettes or vaping devices, have drawn substantial attention recently due to their aggressive marketing, perceived lower toxicity compared to conventional cigarettes, and a surge in popularity among younger populations [1]. Unlike combustible tobacco, which releases thousands of chemicals through combustion, ENDS produce aerosols containing nicotine, volatile organic compounds, formaldehyde, and heavy metals at various concentrations [2,3]. Although advertised as reduced-risk products, the complex chemical composition of ENDS aerosols signals a potential for adverse health effects, including those related to the oral cavity [4,5].

Periodontitis is one of the most prevalent oral diseases, characterized by a persistent inflammatory process that leads to the loss of the supporting tissues around the teeth [6]. Environmental and behavioral risk factors, such as smoking, have consistently been implicated in exacerbating periodontal breakdown, largely by increasing the release of cytokines (e.g., interleukin-1β, interleukin-6, tumor necrosis factor-α) and altering immune cell function [7,8]. Cigarette smoking, in particular, shifts the oral microbiome towards pathogenic taxa, compromises host defenses, and impairs the healing process following periodontal therapy [4,9]. Notably, nicotine is believed to be a key mediator of these negative effects since it can directly modulate neutrophil activation and elastase release, thereby amplifying tissue destruction [10,11].

Although there is strong evidence linking combustible cigarettes to adverse periodontal outcomes, far fewer studies have evaluated the effects of ENDS on periodontal health [12]. Preliminary investigations indicate that some ENDS users exhibit elevated levels of inflammatory biomarkers compared to non-smokers, though these levels appear lower than those observed in traditional cigarette smokers. Likewise, analyses of microbial shifts among vapers suggest an intermediate pattern between non-smokers and combustible cigarette users [13]. However, these findings are hindered by methodological inconsistencies, including variable study designs, participant demographics, and analytical metrics. Moreover, ENDSs encompass a diverse array of devices, each with its own heating coil materials, nicotine concentrations, and flavoring additives, potentially resulting in heterogeneous health outcomes.

From a pathophysiological standpoint, ENDS aerosols may elicit immunomodulatory effects similar to those of traditional cigarettes by triggering oxidative stress, altering fibroblast migration, and promoting the production of pro-inflammatory mediators [12]. The presence of nicotine alone, regardless of its source, could upregulate prostaglandin production and neutrophil activity, aggravating periodontal tissue destruction [2,10]. Additionally, flavoring chemicals and aldehydes in ENDS aerosols may exacerbate inflammatory responses or induce cytotoxic effects in gingival epithelial and periodontal ligament cells [14]. Despite the mounting concerns and conclusive statements on the relative periodontal risks of ENDS versus traditional cigarettes, the issue remains elusive due to the limited volume of long-term research [15].

Understanding whether ENDSs confer a “harm reduction” advantage over conventional cigarettes, particularly in the context of periodontal disease, requires appraisal of clinical parameters, key inflammatory markers, and oral microbial shifts. While systematic reviews and primary studies have examined these parameters associated with ENDS use, individually or in limited combinations, these findings remain fragmented across disciplines. To date, there has been no integrative narrative synthesis that contextualizes periodontal clinical findings alongside evidence from inflammatory response and the microbiome. Therefore, this review aims to synthesize existing evidence from studies assessing ENDS users, cigarette smokers, and non-smokers, integrating periodontal outcomes, inflammatory markers, and oral microbiome changes.

## 2. Materials and Methods

This narrative review was conducted to synthesize the current evidence linking periodontal clinical findings, inflammatory responses, and changes in the oral micro-biome in ENDS users compared with cigarette smokers and non-smokers.

### 2.1. Search Strategy

A comprehensive and structured literature search was conducted in PubMed (MEDLINE), guided by the following PICO question: “What is the impact of ENDS, compared to cigarette smoking and non-smoking, on periodontal health?” This systematic approach was used to reduce selection bias.

PICO:

Population: Subjects aged ≥18 years.
Intervention or Exposure: ENDS users.
Comparison Groups: Cigarette smokers and non-smokers.
Outcomes: Clinical periodontal parameters (bleeding on probing (BoP), plaque index (PI), gingival index (GI), probing depth (PD), and clinical attachment loss (CAL)), as well as levels of inflammatory biomarkers (e.g., interleukin [IL]-1β, IL-6, tumor necrosis factor-alpha [TNF-α]), and alterations in oral microbiome composition.

A combination of Medical Subject Headings (MeSH) terms and free-text keywords, combined with Boolean operators (AND, OR) for maximum sensitivity, was used. The core search string included: (“Electronic Nicotine Delivery Systems” OR “e-cigarettes” OR “vaping”) AND (“oral microbiome” OR “periodontitis” OR “periodontal” OR “gingival”) AND (“inflammation” OR “inflammatory markers” OR “cytokines”). Database-specific filters were applied to limit the publication range (January 2009–May 2026), and no language filters were imposed initially. In addition, the reference lists of the included articles and relevant reviews were manually screened for studies that met our eligibility criteria but were not captured by the initial search.

### 2.2. Eligibility Criteria and Study Selection

We included peer-reviewed original research articles (cross-sectional, case–control, cohort, or interventional studies) published between 1 January 2009, and 1 May 2026, investigating the impact of ENDS and traditional cigarette smoking, compared to no smoking habit, on at least one of the following categories: (1) clinical periodontal parameters, (2) inflammatory response, and (3) oral microbiome. Editorials, conference abstracts, case reports, pilot studies, review articles, and in vitro studies, as well as studies focusing on populations with significant confounding systemic conditions (e.g., uncontrolled diabetes mellitus, autoimmune diseases) that could independently affect periodontal health, were excluded. Lastly, articles not available in English or lacking full-text access were excluded.

Two reviewers (S.P. and J.Y.) independently reviewed the titles and abstracts of all retrieved records. Articles deemed potentially eligible underwent full-text review. During the full-text screening, any discrepancies regarding inclusion were resolved through discussion. The reasons for exclusion at the full-text level (e.g., wrong population, insufficient data on periodontal outcomes) were recorded.

### 2.3. Data Extraction

A standardized data extraction form was developed and pilot-tested on a subset of articles to ensure consistency. The following information was extracted independently by two reviewers (S.P. and J.Y.):

• Study Characteristics: Author(s), publication year, and study design.
• Participant Information: Sample size, age range, and inclusion/exclusion criteria.
• Intervention/Exposure Details: Duration of ENDS use, frequency of use, comparator group details (smoking status, pack-years, or self-reported non-smokers).
• Periodontal Outcomes: BoP, PI, PD, CAL, and any periodontal indices measured.
• Inflammatory Markers: Levels of IL-1β, IL-6, TNF-α, or other relevant cytokines.
• Microbiological Assessments: Composition or diversity of the oral/periodontal microbiome, identification of key bacterial species, or taxa shifts.
• Key Findings: Significant results, statistical analyses, and authors’ conclusions.

Discrepancies in extracted data were resolved through re-examination of the original articles and consensus among the reviewers. Given the heterogeneity of study designs, participant populations, and outcome measures, a quantitative synthesis or meta-analysis was deemed inappropriate.

## 3. Results

### 3.1. Database Search

Following the database search and the systematic screening of the titles, abstracts, and full texts, a total of 10 studies were included in this review. The main reasons for exclusion included (1) irrelevant study design, (2) commentary articles, (3) in vitro studies, (4) lack of access to full text, and (5) classification as systematic reviews.

### 3.2. Risk of Bias

Even though the narrative design of this study does not require a risk-of-bias assessment, we believe this information provides additional insight into the level of scientific rigor of the included studies.

The risk of bias was evaluated using an adapted Newcastle–Ottawa Scale (NOS) for cross-sectional designs [16]. Overall scores ranged from 7 to 9 stars out of a possible 11 (Table 1), with higher scores indicating a lower risk of bias.

All studies received stars for sample representativeness and justification of sample size, although none adequately addressed non-respondent bias. Exposure ascertainment and outcome measurements were consistently strong across studies, with validated or clearly described methods reported. Statistical tests were appropriate and clearly documented.

Comparability of participant groups varied slightly; while most studies adjusted for at least one confounder, two studies [17,18] controlled for multiple confounders and received higher scores. Six studies [19,20,21,22,23] received 8 stars, one study [17] received 9 stars, and three studies [18,24,25] received 7 stars. Despite minor methodological differences, most studies demonstrated a low risk of bias and were included in the qualitative synthesis.

**Table 1:** Newcastle-Ottawa scale of studies included in the review.

Study	Mokeem et al. (2018) [20]	BinShabaib et al. (2019) [21]	Vohra et al. (2020) [22]	Aldakheel et al. (2020) [23]	Ganesan et al. (2020) [17]	Pushalkar et al. (2020) [24]	Xu et al. (2021) [19]	Ali et al. (2022) [26]	Wang et al. (2022) [25]	Thomas et al. (2022) [18]
Selection Representativeness	*	*	*	*	*	*	*	*	*	*
Sample Size	*	*	*	*	*			*		
Non-respondents										
Ascertainment of exposure	**	**	**	**	**	**	**	**	**	**
Comparability	*	*	*	*	**	*	**	*	*	*
Outcome	**	**	**	**	**	**	**	**	**	**
Statistical Test	*	*	*	*	*	*	*	*	*	*
Score	8	8	8	8	9	7	8	8	7	7

0–3 stars: high risk of bias; 4–6 stars: medium risk of bias; 7–9 stars: low risk of bias; 10–11 stars: very low risk of bias.

### 3.3. Study and Participant Characteristics

Of the studies included, the majority were cross-sectional, with a smaller subset comprising observational and case–control designs (Table 2). Sample sizes ranged from as few as 25 participants in smaller studies to as many as 120 participants in larger cohorts. Participant groups typically included cigarette smokers, ENDS users, and non-smokers. ENDS users were characterized by exclusive use for a minimum duration, often ranging from 6 months to several years, while cigarette smokers were required to meet criteria such as smoking a specified number of cigarettes daily over a defined period (e.g., ≥5 cigarettes per day for 1–10 years).

The studies examined diverse outcomes, including clinical periodontal parameters, inflammatory markers, and changes in the oral microbiome. ENDS use patterns varied; some studies detailed the types of devices or e-liquids used, while others focused on frequency and duration of use, such as daily puff counts or years of exclusive vaping. Demographic and behavioral data, such as age, smoking history, and health status, were consistently reported for each group, providing context for comparisons. Definitions for non-smokers and non-ENDS users were also included, but varied across studies.

### 3.4. Periodontal Health Outcomes Across Groups

Results on the evaluation of periodontal parameters are presented in Table 3 and Table 4. The data summarize the clinical periodontal parameters extracted from the included studies and present findings by study type (cross-sectional—Table 3 and observational—Table 4). Results of each study are listed, providing a comparison between included groups.

#### 3.4.1. Plaque Index (PI)

Studies not specifying the periodontal status found that cigarette smokers had a significantly higher plaque index (PI) than both ENDS users and non-smokers [20,21,22]. However, when periodontal status was specified, both cigarette smokers and ENDS users showed higher PI than non-smokers without periodontitis. In patients with periodontitis, PI levels were comparable among cigarette smokers, ENDS users, and non-smokers. These findings suggest that periodontal status influences plaque accumulation and may be a stronger determinant of PI than smoking or vaping status alone.

#### 3.4.2. Gingival Index (GI)

Aldakheel et al. (2020) [23] was the only study to assess GI, finding no significant differences among smokers, ENDS users, and non-smokers with periodontitis, suggesting that periodontal disease may overshadow the effects of nicotine or vaping-related factors on gingival inflammation.

#### 3.4.3. Bleeding on Probing (BoP)

BoP percentages were generally lower in cigarette smokers and ENDS users compared to non-smokers, likely due to nicotine-induced vasoconstriction masking the underlying inflammation [20,21]. However, one study found no significant differences among all groups.

#### 3.4.4. Probing Depth (PD)

Cigarette smokers exhibited deeper pockets compared to ENDS users and non-smokers in studies without specified periodontal status [20,21,22]. However, when periodontal status was specified, both cigarette smokers and ENDS users showed higher PD than non-smokers without periodontitis. At the same time, PD levels were comparable among cigarette smokers, ENDS users, and non-smokers with periodontitis.

#### 3.4.5. Clinical Attachment Loss (CAL)

The findings on CAL suggest that smoking and ENDS use contribute to greater attachment loss, particularly in individuals without periodontitis. Studies have shown that cigarette smokers exhibit significantly higher CAL than ENDS users and non-smokers [20,21]. However, Vohra et al. (2020) [22] found no significant difference in CAL among the groups. Other studies reported that both CS and ES had greater CAL than NS without periodontitis, while CAL levels were comparable among CS, ES, and NS with periodontitis. This suggests that periodontal disease itself may be a stronger determinant of attachment loss than smoking or vaping status alone [23,26].

### 3.5. Inflammatory Markers

#### 3.5.1. ENDS Users Versus Cigarette Smokers

The inflammatory response associated with ENDS use was evaluated relative to cigarette smoking across multiple studies (Table 5), focusing primarily on IL-1β, IL-6, and TNF-α—key cytokines implicated in periodontitis—alongside other markers, including IL-4, IL-8, IFN-γ, and MMP-8.

IL-1β levels were consistently reported as significantly lower in ENDS users compared to cigarette smokers [18,20,21]. An exception was Pushalkar et al. (2020) [24], who observed elevated IL-1β in ENDS users, though the difference was not statistically significant. Similarly, IL-6 was significantly reduced in ENDS users in two studies [20,21] (*p* < 0.01, <0.05), while three other studies reported no significant differences [17,18,24]. These findings suggest a generally attenuated IL-1β- and IL-6-mediated inflammatory response in ENDS users compared to cigarette smokers.

Results for TNF-α levels were more variable. BinShabaib et al. (2019) [21] found significantly lower TNF-α levels in ENDS users (*p* < 0.05), whereas Pushalkar et al. (2020) [24] and Ganesan et al. (2020) [17] reported no significant differences. In contrast, Thomas et al. (2022) [18] observed significantly higher TNF-α levels in ENDS users. These inconsistencies prevent us from drawing a definitive conclusion regarding ENDS-associated TNF-α expression.

Among additional markers, IL-4 was significantly lower in ENDS users in one study [18] (*p* < 0.05), while others [17,24] found no difference. IL-8 was generally unchanged across groups, except in one study [17], in which ENDS users had significantly higher levels. IFN-γ and MMP-8 levels were significantly lower in ENDS users [21] (*p* < 0.05), suggesting less periodontal tissue destruction in ENDS users.

#### 3.5.2. ENDS Users Versus Non-Smokers

The inflammatory profile of ENDS users was also assessed in comparison to non-smokers (Table 6), again focusing on IL-1β, IL-6, and TNF-α. Most studies found no significant difference in IL-1β between ENDS users and non-smokers, suggesting that ENDS use does not markedly elevate this pro-inflammatory cytokine. In contrast, IL-6 results were inconsistent: Ganesan et al. (2020) [17] reported significantly higher IL-6 levels in ENDS users (*p* < 0.05), while other studies [18,20,21,24] showed no difference, rendering its role inconclusive.

TNF-α was consistently elevated in ENDS users in two studies [17,18], indicating a potential for TNF-α–driven inflammation linked to periodontal tissue destruction. Additional markers showed a mixed pro-inflammatory pattern. IL-2 levels were significantly higher in ENDS users. IL-10, an anti-inflammatory cytokine, on the other hand, showed opposite trends among two studies assessing these levels [17,18]. IL-15 and IL-18 were significantly elevated in ENDS users per [26] (*p* < 0.001), while GM-CSF also increased (*p* < 0.05) [17]. MMP-8 showed no significant difference between groups [21]. Notably, IFN-γ levels were significantly higher in ENDS users [17,18] (*p* < 0.05), supporting a heightened inflammatory response.

In summary, ENDS users demonstrated a distinct inflammatory profile compared to non-smokers. Elevations in TNF-α, IL-2, IL-15, IL-18, GM-CSF, and IFN-γ suggest activation of specific pro-inflammatory pathways, whereas the absence of significant changes in IL-1β and IL-6 points to selective immune modulation. These findings underscore the complexity of ENDS-associated inflammation and its potential implications for periodontal health.

### 3.6. Oral Microbiome

#### 3.6.1. ENDS Users Versus Cigarette Smokers

The oral microbiome of ENDS users, compared with that of cigarette smokers, showed distinct compositional changes, indicating unique microbial adaptations to ENDS aerosols. These differences included the enrichment of certain taxa and the suppression of others, highlighting the distinct impacts of vaping versus smoking on microbial ecosystems.

ENDS users showed elevated levels of microbial taxa associated with inflammation and dysbiosis (Table A1):

• Phylum-Level Increases: *Fusobacteria*, consistently more prevalent in ENDS users, reflected a potential association with periodontal inflammation. Proteobacteria, another phylum enriched among ENDS users, may indicate altered environmental conditions in the oral cavity that are conducive to microbial shifts.
• Species-Level Increases: *Candidatus Saccharibacteria TM8* and *Veillonella* species were elevated in ENDS users compared to smokers. These taxa may reflect microbial adaptations to ENDS aerosols, with *Veillonella* species linked to biofilm formation and anaerobic metabolism. However, *Veillonella* displayed mixed trends across studies, with some reporting increases and others decreases, suggesting variability in microbial responses.
• Genus-Level Changes: Genera such as *Abiotrophia and Cardiobacterium* were notably elevated in ENDS users, reflecting a microbial profile less associated with classical smoking-induced dysbiosis. Other genera, including *Leptotrichia, Ottowia, and Parvimonas*, were also more abundant in ENDS users.

ENDS users exhibited reductions in several microbial taxa commonly enriched in smokers:

• Phylum-Level Decreases: *Firmicutes*, a phylum often elevated in smokers, was significantly reduced in ENDS users.
• Genus-Level Decreases: Genera such as *Megasphaera, Scardovia, Stomatobaculum, and Streptococcus* were less abundant in ENDS users. These reductions may reflect differences in the ability of microbial communities to adapt to ENDS aerosol exposure versus cigarette smoke.

#### 3.6.2. ENDS Users Versus Non-Smokers

The comparative analysis of the oral microbiome between ENDS users and non-smokers identified distinct compositional changes, suggesting dysbiotic shifts associated with ENDS use. These changes, characterized by an increase in potentially pathogenic taxa and a decrease in commensal organisms, underscore the impact of ENDS on the oral microbial ecosystem.

ENDS users exhibited notable increases in microbial taxa associated with periodontal disease and dysbiosis (Table A2):

• Species-Level Increases: *Candidatus Saccharibacteria TM8*, an emerging taxon linked to dysbiosis, was also more prevalent in ENDS users. *Veillonella* species showed mixed trends: some studies reported increases in ENDS users, suggesting enhanced biofilm formation, while others reported decreases.
• Genus-Level Changes: Genera such as *Lactobacillus and Parvimonas* were enriched in ENDS users, reflecting their roles in anaerobic metabolism, biofilm formation, and periodontal inflammation.
• Phylum-Level Increases: *Fusobacteria*, a phylum strongly associated with periodontal disease, were elevated in ENDS users compared to non-smokers.

Conversely, ENDS users showed reductions in several health-associated microbial taxa:

• Genus-Level Decreases: *Neisseria* and *Streptococcus* were notably less abundant in ENDS users, potentially reducing microbial resilience and increasing susceptibility to dysbiosis.

## 4. Discussion

This review sought to assess the impact of ENDS use on clinical periodontal parameters, inflammatory markers, and the oral microbiome. Previous reviews have demonstrated that the use of ENDS led to changes in clinical periodontal parameters that were characterized between those of cigarette smokers and non-smokers. These previous observations were consistent with our findings. To our knowledge, this is the first review to evaluate the impact of ENDS use on periodontal health, inflammatory response, and microbial outcomes compared with cigarette smoking and non-smokers.

### 4.1. Clinical Parameters

Clinical periodontal findings across included studies indicate that the effects of ENDS use on clinical parameters are neither uniform nor independent of baseline periodontal status. Rather than demonstrating a consistent exposure-related gradient, clinical parameters appear to be modulated by underlying disease presence, which frequently obscures differences between cigarette smokers, ENDS users, and non-smokers [23,26].

Within this framework, ENDS users presented a unique periodontal deterioration that was significantly more pronounced than that of non-smokers, especially in the cohorts that used ENDS for longer periods [19,21,26]. Some factors that might have contributed to variation in findings include the frequency and intensity of use, variations in inclusion criteria across studies, and the duration of the habit and type of device. ENDS use was associated with clinical findings that differed across studies, presenting its position as a distinct exposure rather than a predictable intermediate along a severity continuum between non-smokers and cigarette smokers.

Plaque accumulation among ENDS users was noted to be higher than among non-smokers but lower than among cigarette smokers [20,23,26]. Studies [17,27,28] have indicated that the glycerol/glycol vehicle in e-liquids acts as a nutrient source, altering biofilm architecture and promoting bacterial adhesion, which might explain the differences observed compared with non-smokers. In the presence of periodontal disease, however, the local inflammatory process appears to negate the effects of ENDS on the plaque index, as studies have demonstrated no differences in this parameter between groups [23,26].

The lower BoP values observed in ENDS users and cigarette smokers, compared to non-smokers, highlight a fundamental limitation of relying on clinical signs of inflammation to assess periodontal health in this cohort. Nicotine-associated suppression of gingival bleeding, through its vasoconstrictive effects, may further obscure ongoing inflammatory or immunologic activity, creating a dissonance between clinical appearance and underlying tissue response [29,30]. These results, when correlated with data on inflammatory markers, suggest that ENDS may increase the inflammatory burden contributing to the observed signs of clinical attachment loss. Collectively, these findings highlight that clinical parameters may underestimate periodontal risk in ENDS users, particularly when disease identification is incomplete or absent, underscoring the importance of immunological and microbial data in evaluating the periodontal implications of ENDS use. Clinicians should therefore interpret low clinical inflammation markers, such as BoP, with caution, recognizing that they do not necessarily reflect healthy periodontal tissues in patients who smoke or vape.

The literature on cigarette smoking and periodontal disease has identified a clear dose–response relationship where greater duration and higher intensity of smoking are independently associated with worse periodontal destruction [31,32]. Information on the impact of ENDS on periodontal disease has increased recently; however, there is yet to be conclusive evidence regarding the effects of ENDS duration and intensity on periodontal disease. Of the studies included in this review, Ali et al. (2022) [26] was the only one to include subjects with a long-term history of ENDS use (12.5 years). However, others [17,18] emphasized that the duration of the habit was a strong source of variation among the studied groups. Therefore, future studies should focus on evaluating the impact of the duration of habit and the intensity of ENDS use on periodontal disease severity and outcomes, as it has been demonstrated with cigarette smoking.

### 4.2. Inflammatory Response

The analysis of inflammatory markers offers valuable insight into the immunological effects of ENDS use relative to both cigarette smoking and non-smoking. Overall, ENDS users exhibited a distinct inflammatory profile, often falling between cigarette smokers and non-smokers, though notable variability across studies highlights the complexity of these interactions.

ENDS users consistently exhibited lower levels of IL-1β and IL-6, cytokines central to periodontal connective tissue breakdown and osteoclast activation, compared with cigarette smokers [33,34,35,36]. This attenuation may reflect the absence of combustion-derived toxicants present in cigarette smoke, which are known to amplify NF-kB-mediated inflammatory signaling [36,37]. This finding supports prior evidence that cigarette smoke induces a broader and more potent inflammatory response than nicotine exposure alone [38,39,40]. Compared with non-smokers, ENDS users more consistently showed elevations in cytokines associated with cell-mediated immunity, including TNF-α, IFN-γ, IL-2, IL-15, and IL-18. As these cytokines are primarily involved in cellular immune responses characteristic of chronic inflammation, this pattern suggests a shift toward sustained immune activation rather than acute innate inflammation [41,42]. The relative stability of IL-1β and IL-6 may indicate a less intense inflammatory response in ENDS users compared to cigarette smokers; however, the observed cytokine profile in ENDS users suggests chronic immune activation, which may have implications for periodontal tissue homeostasis over time [43,44]. Importantly, this immune activation may occur in the absence of overt clinical inflammation, highlighting the limitations of relying solely on clinical parameters.

Variations in sampling technique were noted in the included studies. Some studies used unstimulated whole saliva [20,26], whereas others collected gingival crevicular fluid from sites with the highest disease severity [17,21]. These variations in sampling techniques can affect cytokine concentrations and lead to an unfair comparison between results. More specifically, if one samples directly from a periodontal pocket, the concentrations will be much higher than those measured in whole saliva. Therefore, we recommend caution when comparing results derived from studies presenting different sampling methodologies.

Collectively, the findings indicate that while ENDS use may be associated with a milder inflammatory profile compared to cigarette smoking, it is not without immunological consequences. Compared to non-smokers, ENDS users exhibited several elevated inflammatory markers, pointing toward a distinct and potentially pathogenic immune response. Given the chronic nature of periodontitis and its reliance on sustained inflammation, these immune shifts could have long-term clinical implications, particularly in frequent or long-term ENDS users.

### 4.3. Oral Microbiome Shifts

The oral microbiome is a dynamic ecological system that contributes to immune regulation and periodontal homeostasis, with microbial composition varying substantially across distinct oral niches [45,46]. Disruption of this equilibrium may promote dysbiosis associated with periodontal inflammation and tissue breakdown [47,48]. Within this context, available evidence indicates that ENDS use is associated with measurable alterations in oral microbial composition that do not consistently replicate profiles observed in either cigarette smokers or non-smokers. Rather than occupying a predictable intermediate position, ENDS users exhibited variable shifts in microbial composition, including enrichment of taxa linked to periodontal inflammation, such as *Tannerella forsythia*, *Fusobacteria*, and *Bacteroidetes*, and reduction in health-associated commensals, including *Neisseria, Streptococcus*, and *Actinomyces* [49,50,51,52]. Notably, these alterations have been reported even in the absence of consistent differences in traditional clinical periodontal parameters, suggesting that microbial perturbations may occur independently of, or precede, overt clinical manifestations [44,53,54]. The dysbiotic profile observed in ENDS users is directly linked to the clinical signs of periodontal disease reported in the studies, where a strong positive correlation among periodontal pathogens, proinflammatory cytokines, and clinical signs of disease was observed [17,18,24]. Overall, ENDS use appears to promote a distinct, potentially pathogenic microbial environment that differs from both traditional smoking and non-use, warranting further research into its long-term implications for oral and periodontal health.

Evidence from the reviewed studies underscores significant inconsistencies in the reported oral microbiome shifts among ENDS users compared to smokers and non-smokers. These discrepancies arise from several intertwined factors. Sampling methodology should be carefully noted, as studies applied not only different biofilm collection methods, but also different collection sites (supragingival versus subgingival). On the same token, detection methods (16S sequencing versus cultures) also differed between studies, and this might be one of the most significant factors driving differences in results. It is also important to note that differences in ENDS device design, power settings, e-liquid components (including flavorings and nicotine concentration), and user habits (frequency, duration, or dual use with combustible cigarettes) further complicate the delineation of ENDS-specific microbial signatures. Lastly, the oral microbiome is modulated by host factors such as immune competence, dietary patterns, and systemic health, which are not always uniformly accounted for in the study protocols. Despite these sources of variability, the overall trajectory indicates that ENDS use can induce measurable shifts in the oral microbiome, including increased abundances of putative pathogens (e.g., *Tannerella forsythia, Treponema*) and reduced commensal populations (e.g., *Neisseria*). These changes may foster an anaerobic, pro-inflammatory environment conducive to periodontal breakdown.

### 4.4. Limitations of Included Studies

The present review does not generate pooled estimates; instead, it integrates periodontal clinical findings with evidence from inflammation and the oral microbiome, which have largely been evaluated separately in prior reviews. Several limitations have been reported and discussed previously; however, it is important to note a few other factors that might have contributed to the heterogeneity of the data presented.

Most included studies are observational [19] and cross-sectional [17,18,20,21,22,23,24,25,26] in design, which limits causal inference and increases susceptibility to residual confounding. A major challenge in interpreting ENDS-related outcomes is the high proportion of former cigarette smokers, which complicates the attribution of findings specifically to ENDS exposure, even when current dual users are excluded. Although the present review did not evaluate data from dual users, variability in prior smoking history, reliance on self-reported exposure without biochemical verification, and heterogeneity in exposure definitions limit the overall quality of evidence. While several previously published systematic reviews and meta-analyses quantitatively synthesized outcomes associated with ENDS use [55,56,57], these analyses were similarly constrained by substantial heterogeneity and reliance on observational data, necessitating the cautious interpretation of pooled effect estimates. Additional heterogeneity arises from variability in device characteristics, e-liquid composition (including flavoring and nicotine concentrations), and usage patterns (frequency and duration), as well as inconsistencies in exposure definitions and outcome measures across studies. Methodological differences, including variation in sampling sites (saliva versus supra- or subgingival plaque) and in sample processing protocols (timing of collection relative to ENDS/cigarette use or oral hygiene practices), further limit comparability.

## 5. Conclusions

This narrative review synthesizes current evidence on the impact of ENDS use on periodontal health, integrating clinical periodontal parameters, inflammatory biomarkers, and oral microbiome findings. Across included studies, ENDS use was associated with alterations in inflammatory profiles and oral microbiome composition that differed from both cigarette smoking and non-use, underscoring ENDS as a distinct exposure rather than a predictable intermediate along a smoking continuum. While these biological changes were not consistently reflected in clinical parameters, their presence suggests potential subclinical or context-dependent effects with relevance to periodontal tissue homeostasis.

Given the increasing prevalence of ENDS use, particularly among younger populations, oral healthcare providers should be aware of the potential biological effects associated with vaping. Patient education about the effects of ENDS on periodontal health, as well as close monitoring of patients’ oral health, will be fundamental to preventing deterioration of periodontal health. The incorporation of inflammatory and microbial assessments alongside traditional clinical assessment may improve periodontal risk evaluation in ENDS users.

Future research should focus on well-designed observational studies that incorporate larger cohorts, detailed profiling of ENDS devices and e-liquid formulations, and rigorous control of host-related confounders. Such efforts will be crucial in clarifying the clinical implications of ENDS-associated microbial dysbiosis on oral and periodontal health.

## Figures and Tables

**Table 2:** Study characteristics divided into author/year, study design, inclusion criteria, cohort and habit characteristics, and type of data collected.

Author/Year	Study Design	Inclusion Criteria			Cohort Characteristics	Habit Characteristics	Data Collected on
		Cigarette Smoker	ENDS User	Non-Smoker			
**Mokeem et al. (2018)** [20]	Cross-sectional	Individuals who reported smoking at least 5 cigarettes daily for at least 12 months	Individuals who reported vaping exclusively e-cigarettes for at least 12 months and had never used smoked tobacco in the past	Individuals who reported having never used smoked tobacco and/or consumed smokeless tobacco products	39 cigarette smokers, 37 ENDS users and 38 never smokers with mean ages of 42.4 ± 5.6, 29.3 ± 1.6, and 40.6 ±4.5 years, respectively	Cigarette smokers: 16.2 ± 4.5 times daily for 17.2 + 2.5 years ENDS users: 9.2 ± 1.4 times daily for 3.1 ± 0.4 years	Clinical periodontal findings, inflammatory markers
**BinShabaib et al. (2019)** [21]	Cross-sectional	Individuals who smoked ≥5 cigarettes daily for at least 1 year	Individuals exclusively using e-cigarettes at least once daily	Individuals who reported to have never used any form of tobacco product	44 cigarette smokers, 44 ENDS users and 45 never smokers with mean ages of 36.5 ± 1.7, 44.2 ± 3.5, and 40.6 ± 3.3 years, respectively	Cigarette smokers: 14.2 ± 0.6 pack years ENDS users: 9.4 ± 2.6 years of use	Clinical periodontal findings, inflammatory markers
**Vohra et al. (2020)** [22]	Cross-sectional	Individuals who smoked 1 pack or up to 20 cigarettes daily	Individuals using e-cigarette/JUUL at least once daily as the sole source of nicotine intake	Individuals who reported to have never used tobacco in any form	28 cigarette smokers, 26 electronic cigarette users, 25 JUUL users, and 26 never smokers with mean ages of 33.3 ± 2.2, 31.6 ± 2.4, 32.1 ± 1.7 and 33.5 ± 1.4 years, respectively	Cigarette smokers: 6.1 ± 0.5 pack years E-cigarette users: 30.2 ± 8.5 times/day for 0.9 ± 0.2 years JUUL users: 25.3 ± 6.4 times/day for 0.8 ± 0.1 years	Clinical periodontal finding
**Aldakheel et al. (2020)** [23]	Cross-sectional	Individuals who smoked at least 5 cigarettes daily for past 12 months.	Individuals exclusively vaping at least once daily for past 12 months	Individuals who reported to have never used any form of tobacco product	15 cigarette smokers, 15 ENDS users, 15 non-smokers with periodontitis and 15 non-smokers without periodontitis with mean ages of 40.5 ± 2.1, 38.6 ± 3.3, 39.4 ± 1.6 and 39.5 ± 0.8 years, respectively	Cigarette smokers: 10.4± 0.8 pack years ENDS users: formers cigarette smokers solely vaping for 3.3 ± 0.2 years	Clinical periodontal findings, oral microbiome
**Ganesan et al. (2020)** [17]	Cross-sectional	Individuals who smoked ≥10 cigarettes daily for at least 5 years	Individuals exclusively using e-cigarettes daily for at least 6 months	Individuals who have never used any form of tobacco or e-cigarettes	25 cigarette smokers, 20 ENDS users, 25 non-smokers with mean ages of 25 ± 5, 23 ± 4, 22 ± 3, respectively	Cigarette smokers: 10 ± 2 pack years ENDS users: 15 ± 3 times/day	Inflammatory markers, Oral microbiome
**Pushalkar et al. (2020)** [24]	Cross-sectional	Individuals who smoked ≥10 cigarettes daily for at least 5 years	Individuals exclusively using e-cigarettes daily for at least 6 months	Individuals who have never used any form of tobacco or e-cigarettes	40 cigarette smokers, 40 ENDS users, 39 non-smokers with mean ages of 42.5 ± 6.3, 39.2 ± 7.1, 38.4 ± 6.6, respectively	Cigarette smokers: At least 10 cigarettes/day ENDS users: 0.5–1 e-cigarettes/day	Oral microbiome, inflammatory markers
**Xu et al. (2021)** [19]	Observational	Individuals who smoked at least 10 cigarettes per day for 12 months or more	Individuals who use a minimum of 0.5–1 e-cigarette daily for the last 6 months	Individuals who have never smoked a cigarette or used an e-cigarette in their lifetime	31 cigarette smokers, 32 ENDS users, and 38 non-smokers with mean male ages of 46.9 ± 10.1, 36.0 ± 9.8, 28.8 ± 6.1 and mean female ages of 46.6 ± 11.5, 38.7 ± 10.6, 39.0 ± 14.1, respectively	Cigarette smokers: At least 10 cigarettes/day ENDS users: 0.5–1 e-cigarettes/day	Clinical Periodontal findings
**Ali et al. (2022)** [26]	Case–control	Individuals who currently smoked and had smoked at least 100 cigarettes in their lifetime	Individuals who had used ENDS at least once in the past 30 days	Individuals who have never consumed combustible or non-combustible nicotinic products	19 cigarette smokers, 18 ENDS users, 19 never smokers with periodontitis and 19 never smokers without periodontitis with mean ages of 52.6 ± 6.1, 49.5 ± 2.3, 50.7 ± 2.2 and 48.1 ± 1.3 years, respectively	Cigarette smokers: 24.3 ± 0.7 pack years ENDS users: 25.1 ± 3.5 times daily with 6.6 ± 0.7 puffs per session for 12.5 ± 0.8 years	Clinical periodontal findings, inflammatory markers
**Wang et al. (2022)** [25]	Cross-sectional	Individuals who smoked cigarettes daily for at least 5 years	Individuals using e-cigarettes exclusively for a minimum of 6 months	Individuals who have never used any form of tobacco or e-cigarettes	14 cigarette smokers, 5 ENDS users, 6 non-smokers with mean ages of 37.52 ± 9.19, 36.18 ± 11.62, 41.33 ± 12.21, respectively	Cigarette smokers: 5 pack years ENDS users: N/A	Oral microbiome
**Thomas et al. (2022)** [18]	Observational	Individuals who smoked cigarettes for at least 10 years	Individuals using ENDS exclusively for at least 5 years	Individuals who have never used tobacco products, including ENDS	27 cigarette smokers, 28 ENDS users, 29 non-smokers with mean male ages of 48.2 ± 9.51, 35.8 ± 10.3, 28.6 ± 7.05, and mean female ages of 51 ± 10.3, 39.7 ± 11.3, 38.9 ± 14.9, respectively	Cigarette smokers: 9.3 ± 4.6. pack years ENDS users: 12.5 ± 0.8 puffs per day for 3.1 ± 0.4 years	Oral microbiome, inflammatory markers

**Table 3:** Extracted results on clinical periodontal data from cross-sectional studies.

Author	PI (% of Sites)	GI	BoP (% of Sites)	PD	CAL
**Mokeem et al. (2018)** [20]	CS > ES, NS *	N/A	NS > CS, ES *	CS > ES, NS *	CS > ES, NS *
**BinShabaib et al. (2019)** [21]	CS > ES, NS *	N/A	NS > CS, ES *	CS > ES, NS *	CS > ES, NS *
**Vohra et al. (2020)** [22]	CS > ES, NS *	N/A	No significant difference between groups	CS > ES, NS *	No significant difference between groups
**Aldakheel et al. (2020)** [23]	CS, ES > NS without periodontitis ^‡^ CS~ES~NS with periodontitis	CS, ES > NS without periodontitis ^‡^ CS ~ ES ~ NS with periodontitis	N/A	CS, ES > NS without periodontitis ^‡^ CS~ES~NS with periodontitis	CS, ES > NS without periodontitis ^‡^ CS~ES~NS with periodontitis
**Ali et al. (2022)** [26]	CS, ES > NS without periodontitis ^‡^ CS~ES~NS with periodontitis	CS, ES > NS without periodontitis ^‡^ CS ~ ES ~ NS with periodontitis	N/A	CS, ES > NS without periodontitis ^‡^ CS~ES~NS with periodontitis	CS, ES > NS without periodontitis ^‡^ CS~ES~NS with periodontitis

CS = cigarette smoker, ES = ENDS user, NS = non-smoker; * *p* < 0.05, ^‡^ *p* < 0.001.

**Table 4:** Extracted results on clinical periodontal data from observational studies.

Author/Year	Timepoint	PI (% of Sites)	GI	BoP (% of Sites)	PD	CAL
**Xu et al. (2021)** [19]	Baseline	N/A	N/A	No significant difference between groups	CS > NS ^†^ CS~ES ES~NS	CS, ES > NS ^†^
	6 month follow up	N/A	N/A	No significant difference between groups Increase in all groups but change not statistically different between groups	CS > NS ^†^ Increased significantly in all groups ^‡^ but the increase was comparable across groups	CS, ES > NS ^†^ Increased significantly in ENDS users over 6 months ^†^

CS = cigarette smoker, ES = ENDS user, NS = non-smoker; ^†^ *p* < 0.01, ^‡^ *p* < 0.001.

**Table 5:** Inflammatory markers in ENDS users compared to cigarette smokers.

Inflammatory Marker	Mokeem et al. (2018) [20]	BinShabaib et al. (2019) [21]	Ganesan et al. (2020) [17]	Pushalkar et al. (2020) [24]	Ali et al. (2022) [26]	Thomas et al. (2022) [18]
GM-CSF	N/A	N/A	NS	N/A	N/A	N/A
IL-1β	↓ ^†^	↓ *	N/A	NS	N/A	↓ *
IL-2	N/A	N/A	NS	NS	N/A	NS
IL-4	N/A	N/A	NS	NS	N/A	↓ ^‡^
IL-6	↓ ^†^	↓ *	NS	NS	N/A	NS
IL-8	N/A	N/A	↓ *	NS	N/A	NS
IL-10	N/A	N/A	↑ *	NS	N/A	NS
IL-12p70	N/A	N/A	N/A	NS	N/A	NS
IL-13	N/A	N/A	N/A	NS	N/A	NS
IL-15	N/A	N/A	N/A	N/A	NS	N/A
IL-18	N/A	N/A	N/A	N/A	NS	N/A
IFN-γ	N/A	↓ *	NS	NS	N/A	N/A
MMP-8	N/A	↓ *	N/A	N/A	N/A	N/A
TNF-α	N/A	↓ *	NS	NS	N/A	↑ ^†^

↑ = higher expression in ENDS users, ↓ = lower expression in ENDS users. * *p* < 0.05,^†^ *p* < 0.01, ^‡^ *p* < 0.001, NS: not significant. GM-CSF: granulocyte-macrophage colony-stimulating factor, IL: interleukin, IFN: interferon, TNF: tumor necrosis factor, MMP: Matrix metalloproteinase.

**Table 6:** Inflammatory markers in ENDS users compared to non-smokers.

Inflammatory Marker	Mokeem et al. (2018) [20]	BinShabaib et al. (2019) [21]	Ganesan et al. (2020) [17]	Pushalkar et al. (2020) [24]	Ali et al. (2022) [26]	Thomas et al. (2022) [18]
GM-CSF	N/A	N/A	↑ *	N/A	N/A	N/A
IL-1β	NS	NS	N/A	NS	N/A	NS
IL-2	N/A	N/A	↑ *	NS	N/A	↑ ^†^
IL-4	N/A	N/A	NS	NS	N/A	NS
IL-6	NS	NS	↑ *	NS	N/A	NS
IL-8	N/A	N/A	NS	NS	N/A	NS
IL-10	N/A	N/A	↓ *	NS	N/A	↑ *
IL-12p70	N/A	N/A	N/A	NS	N/A	NS
IL-13	N/A	N/A	N/A	NS	N/A	NS
IL-15	N/A	N/A	N/A	N/A	↑ ^‡^	N/A
IL-18	N/A	N/A	N/A	N/A	↑ ^‡^	N/A
IFN-γ	N/A	NS	↑ *	N/A	N/A	↑ *
MMP-8	N/A	NS	N/A	N/A	N/A	N/A
TNF-α	N/A	NS	↑ *	NS	N/A	↑ ^‡^

↑ = higher expression in ENDS users, ↓ = lower expression in ENDS users. * *p* < 0.05, ^†^ *p* < 0.01, ^‡^ *p* < 0.001. NS: not significant. GM-CSF: granulocyte-macrophage colony-stimulating factor, IL: interleukin, IFN: interferon, TNF: tumor necrosis factor, MMP: Matrix metalloproteinase.

## Data Availability

No new data were created or analyzed in this study. Data sharing is not applicable to this article.

## Appendix A

**Table A1:** Oral microbiome in ENDS users compared to cigarette smokers.

Name	Taxonomy Rank	Aldakheel F et al. (2020) [23]	Ganesan S et al. (2020) [17]	Pushalkar S et al. (2020) [24]	Thomas S et al. (2022) [18]	Wang X et al. (2022) [25]
*Aggregatibacter actinomycetemcomitans*	species	↑				
*Abiotrophia*	genus		↑			
*Actinobacteria*	phylum			↑		
*Aggregatibacter*	genus		↑		↓	
*Bacteroidales*	order				↑	
*Candidatus Saccharibacteria TM8*	species		↑			
*Cardiobacterium*	genus		↑			
*Eikenella*	genus		↑			
*Firmicutes*	genus			↓		
*Fretibacterium*	genus				↓	
*Fusobacteria*	phylum			↑	↑	
*Gemella*	genus				↓	
*Gracilibacteria (GN02)*	genus				↓	
*Granulicatella*	genus		↑		↓	
*Haemophilus*	genus		↑		↓	
*Johnsenella*	genus		↑			
*Kingella*	genus		↑		↓	
*Lachnoanaerobaculum*	genus		↑			
*Lactobacillus*	genus				↑	
*Lautropia*	genus		↑			
*Leptotrichia*	genus		↑		↑	
*Megasphaera*	genus				↓	
*Mogibacterium*	genus		↑			
*Neisseria*	genus				↓	↓
*Oribacterium*	genus				↑	
*Ottowia*	genus		↑			
*Parvimonas*	genus		↑			
*Peptostreptococcus*	genus		↑			
*Prevotella*	genus			↓		↑
*Porphyromonas*	genus				↓	
*Proteobacteria*	phylum			↓		
*Rhodobacter*	genus		↑			
*Rothia*	genus		↑			
*Scardovia*	genus				↓	
*Selenomonas*	genus		↑			
*Stomatobaculum*	genus				↓	
*Streptococcus*	genus				↓	
*Tannerella*	genus				↑	
*Tannerella forsythia*	species	↑				
*Veillonella*	genus		↑	↓		

↑ = higher expression in ENDS users, ↓ = lower expression in ENDS users.

**Table A2:** Oral microbiome in ENDS users compared to non-smokers.

Name	Taxonomy Rank	Aldakheel F et al. (2020) [23]	Ganesan S et al. (2020) [17]	Pushalkar et al. (2020) [24]	Thomas S et al. (2022) [18]	Wang X et al. (2022) [25]
*Abiotrophia*	genus		↑			
*Aggregatibacter*	genus		↑			
*Bacteroidales*	order				↑	
*Candidatus Saccharibacteria TM8*	genus		↑			
*Cardiobacterium*	genus		↑		↑	
*Eikenella*	genus		↑			
*Firmicutes*	phylum			↓		
*Fusobacteria*	phylum			↑	↑	
*Granulicatella*	genus		↑			
*Haemophilus*	genus		↑			
*Johnsenella*	genus		↑			
*Kingella*	genus		↑		↓	
*Lachnoanaerobaculum*	genus		↑			
*Lactobacillus*	genus				↑	
*Lautropia*	genus		↑		↑	
*Leptotrichia*	genus		↑			
*Megasphaera*	genus				↓	
*Mogibacterium*	genus		↑			
*Neisseria*	genus				↓	
*Ottowia*	genus		↑		↑	↓
*Parvimonas*	genus		↑		↑	
*Peptostreptococcus*	genus		↑			
*Prevotellaceae*	genus			↓		
*Proteobacteria*	phylum			↑		↑
*Pseudopropionibacterium*	genus				↑	
*Rhodobacter*	genus		↑			
*Rothia*	genus		↑	↓		
*Scardovia*	genus				↓	
*Selenomonas*	genus		↑			
*Stomatobaculum*	genus				↓	
*Streptococcus*	genus			↓	↓	
*Tanneralla*	genus				↑	
*Tannerella forsythia*	species	↑				
*Veillonella*	genus		↑		↓	

↑ = higher expression in ENDS users, ↓ = lower expression in ENDS users.
